# Postoperative fluid restriction to prevent delayed hyponatremia after endoscopic transsphenoidal surgery

**DOI:** 10.1093/neuonc/noaf069

**Published:** 2025-03-14

**Authors:** Doriann Klaassen, Shinghei Mok, Jenie Y Hwang, Sydney L Blount, Kelley J Williams, Brendan M Fong, Michael R Chicoine, Ralph G Dacey, Nyssa F Farrell, Joshua W Osbun, Keith M Rich, Lauren T Roland, John S Schneider, Gregory J Zipfel, Chongliang Luo, Albert H Kim, Julie M Silverstein

**Affiliations:** Division of Endocrinology, Metabolism, and Lipid Research, Washington University School of Medicine, St. Louis, Missouri, USA; Department of Neurological Surgery, Washington University School of Medicine, St. Louis, Missouri, USA; Department of Pathology and Laboratory Medicine, Emory University School of Medicine, Atlanta, Georgia, USA; Department of Neurological Surgery, Washington University School of Medicine, St. Louis, Missouri, USA; Division of Diabetes, Endocrinology and Metabolism, and Metabolism, University of Nebraska Medical Center, Omaha, Nebraska, USA; Division of Endocrinology, Metabolism, and Lipid Research, Washington University School of Medicine, St. Louis, Missouri, USA; Department of Neurosurgery, Neurosurgery of Saint Louis, St. Louis, Missouri, USA; Department of Neurological Surgery, University of Missouri-Columbia, Columbia, Missouri, USA; Department of Neurological Surgery, Washington University School of Medicine, St. Louis, Missouri, USA; Department of Otolaryngology-Head and Neck Surgery, Washington University in St Louis, St. Louis, Missouri, USA; Department of Neurological Surgery, Washington University School of Medicine, St. Louis, Missouri, USA; Department of Neurological Surgery, Washington University School of Medicine, St. Louis, Missouri, USA; Department of Otolaryngology-Head and Neck Surgery, Washington University in St Louis, St. Louis, Missouri, USA; Department of Otolaryngology-Head and Neck Surgery, Washington University in St Louis, St. Louis, Missouri, USA; Department of Otolaryngology-Head and Neck Surgery, Washington University in St Louis, St. Louis, Missouri, USA; Department of Neurological Surgery, Washington University School of Medicine, St. Louis, Missouri, USA; Division of Public Health Sciences, Washington University School of Medicine, St. Louis, Missouri, USA; The Brain Tumor Center, Siteman Cancer Center, Washington University School of Medicine, St. Louis, Missouri, USA; Department of Neurological Surgery, Washington University School of Medicine, St. Louis, Missouri, USA; The Brain Tumor Center, Siteman Cancer Center, Washington University School of Medicine, St. Louis, Missouri, USA; Department of Neurological Surgery, Washington University School of Medicine, St. Louis, Missouri, USA; Division of Endocrinology, Metabolism, and Lipid Research, Washington University School of Medicine, St. Louis, Missouri, USA

**Keywords:** endoscopic endonasal transsphenoidal surgery, fluid restriction, hyponatremia, pituitary adenoma, pituitary neuroendocrine tumor

## Abstract

**Background:**

Readmission following endoscopic endonasal transsphenoidal surgery (EETS) for pituitary neuroendocrine tumor (PitNET) and other sellar pathology is most commonly due to delayed hyponatremia. Studies suggest postoperative fluid restriction (FR) reduces delayed hyponatremia. We present a prospective randomized controlled study evaluating post-EETS FR.

**Methods:**

300 participants were scheduled for EETS (2016–2023) at a single institution. Patients with CKD, CHF, arginine vasopressin deficiency on postoperative day (POD) 3, chronic hyponatremia, and untreated adrenal insufficiency or hypothyroidism were excluded. Groups included control (ad-lib, *n* = 94), moderate FR (1.8 L/day or 2 L/day weight > 100 kg, *n* = 39), and strict FR (1 L/day or 1.2 L/day weight > 100 kg, *n* = 62) from POD 3–14. Incidence of overall, moderate, and severe hyponatremia (Na < 135, 125–129, and <125 mEq/L), readmission rates, fluid intake, and thirst were evaluated.

**Results:**

The incidence of overall hyponatremia was 31.9%, 28.2%, and 21.0% in control, moderate FR, and strict FR groups, and the incidence of severe hyponatremia was 7.4%, 5.1%, and 0% in control, moderate FR, and strict FR groups. Nadir Na level was higher (1.81 mEq/L; 95% CI, 0.34 to 3.27; *P* = .02) and severe hyponatremia occurred less frequently (95% CI, 0.00 to 1.02; *P* = .04) in the strict FR vs. control group. Readmission was lower in the strict FR (1.6%, *n* = 1) vs. control group (6.4%, *n* = 6).

**Conclusions:**

Postoperative FR decreases rates of delayed hyponatremia and related readmission compared to patients drinking ad-lib. Further studies are needed to assess the optimal volume and duration of FR after EETS.

**Trial registration number:** NCT03636568

**Key Points**

- This randomized controlled trial tests the efficacy of postoperative fluid restriction (FR) to prevent delayed hyponatremia after endoscopic transsphenoidal surgery.
- FR prevents delayed hyponatremia after surgery for PitNETs/other sellar lesions.

**Importance of the Study**

Delayed hyponatremia is the leading cause of 30-day readmission after endoscopic transsphenoidal surgery for pituitary neuroendocrine tumor (PitNET, also pituitary adenoma), the second most common primary brain tumor. Fluid restriction has been proposed as a prophylactic measure to prevent delayed hyponatremia and related readmission. However, the previous literature has been predominantly retrospective in nature, leading to difficulty in assessing the causality of this intervention. This is the first randomized controlled trial to determine the effectiveness of postsurgical fluid restriction on preventing delayed hyponatremia for patients with PitNET and other sellar pathologies following endoscopic endonasal transsphenoidal surgery. Postsurgical fluid restriction demonstrated sustained effectiveness in preventing hyponatremia and related readmission. Thirst assessment suggested that fluid restriction was well-tolerated among patients. In addition, we found fluid restriction to be safe for participants with transient arginine vasopressin deficiency (central diabetes insipidus).

Pituitary neuroendocrine tumors (PitNET, also pituitary adenoma) are among the most common brain tumors^1^ and are estimated to affect 1 in 6 individuals.^2^ Endoscopic endonasal transsphenoidal surgery (EETS) is typically performed for tumors that grow or cause symptoms, such as visual deficits or endocrine disturbances. Importantly, after surgery for these tumors, delayed hyponatremia is the leading cause for 30-day readmission and occurs in up to 35% of patients,^3–5^ most frequently between postoperative day (POD) 7 and 9.^4,6–8^ Delayed hyponatremia accounts for up to 59% of all readmissions following EETS.^9^ Severe cases of hyponatremia can lead to symptoms such as headache, nausea/vomiting, seizures,^6,10,11^ and even death.^12^ The mechanism of hyponatremia following pituitary surgery is not fully understood but felt most likely to be due to syndrome of inappropriate secretion of antidiuretic hormone (SIADH) caused by stalk manipulation,^13–15^ although untreated secondary adrenal insufficiency,^16–18^ desmopressin over-administration, and hypotonic fluid infusion,^7^ and, potentially, dry mouth secondary to nasal packing (and thus oral breathing)^19^ may also be contributing factors. Treatment options include fluid restriction (FR) ± salt tablets^20,21^ and either hypertonic saline^20,22^ or vasopressin receptor antagonists for severe cases.^23^

Existing studies on patient predictors of hyponatremia following EETS have shown conflicting results. Therefore, identifying practical and patient-friendly preventive measures is critical for minimizing the incidence of delayed hyponatremia after pituitary surgery. Several retrospective studies have demonstrated that implementing FR of varying amounts and duration after pituitary surgery decreases rates of delayed hyponatremia from 12–38% to 3–14%^8,24–27^ and readmission from 3–8% to 0–2.4%.^24,27–29^ In a nonrandomized, single-arm, prospective, quality improvement initiative, Cooper et al. demonstrated that the incidence of delayed hyponatremia in 110 patients was reduced from 28% to 12.7% (relative risk (RR) = 0.52), whereas readmissions were reduced from 15% to 4.6% (RR = 0.30) compared to a retrospective control group.^30^ Based on a meta-analysis of 5 retrospective studies with a pooled cohort of 594 patients on FR protocols ranging from 1 to 2.5 L between POD 1 to 15 and 992 control patients, patients on FR had a decreased risk of hyponatremia (RR = 0.34) and readmission due to hyponatremia (RR = 0.24).^5^

Due to limitations inherent to retrospective and single-arm prospective studies,^5,31^ we conducted a prospective randomized controlled trial comparing ad-lib vs. restrictive approaches of postoperative fluid management to test the hypothesis that patients treated with early postoperative FR would have decreased rates of delayed hyponatremia. We also evaluated patient compliance with a FR protocol.

## Materials and Methods

### Trial Design and Participants

Any adult patient with a nonfunctioning or functioning PitNET, Rathke’s cleft cyst, or other pituitary pathology scheduled to undergo EETS at Barnes-Jewish Hospital between August 2016 and February 2023 was screened. Patients without an intact thirst mechanism, with chronic kidney disease stage III or greater, New York Heart Association class heart failure III or IV, history of SIADH (except if secondary to hypothyroidism or adrenal insufficiency, or in association with prior EETS), chronic hyponatremia, or untreated adrenal insufficiency or hypothyroidism were excluded. Patients with arginine vasopressin deficiency (AVP-D) as of POD 3 were also excluded.

Before April 2019, patients were randomized in a 1:1 fashion to a moderate FR protocol (1.8 L per day for patients who weigh ≤ 100 kg and 2 L per day for patients who weigh > 100kg) or control ad-lib group. We planned to recruit a total of 300 patients, with interim analysis planned for the first 100 patients, by a biostatistician unblinded to the allocation of the intervention. This interim calculation of nadir serum sodium (Na) in the moderate FR group indicated that target enrollment with the current FR intervention would lack adequate power (see Statistical Analysis). Along with retrospective studies supporting a stricter FR,^28,29^ the protocol was thus changed after April 2019 to a strict FR protocol (1 L per day for patients who weigh ≤ 100 kg and 1.2 L per day for patients who weigh > 100kg), with participants being randomized in a 2:1 fashion after that point. All patients were started on postoperative weight-based intravenous fluids until POD 1 and then allowed to drink freely. Patients in both FR groups were instructed to start restricting fluids from POD 3 until POD 14, and patients in the control group were instructed to drink fluid ad-lib. All patients in the study had an assessment of their hypothalamic pituitary adrenal (HPA) axis before surgery with an 8:00 A.M. cortisol and/or standard high-dose cosyntropin stimulation test. Patients with adrenal insufficiency were treated with perioperative stress dose steroids. Patients with an intact HPA axis had their cortisol checked at 8:00 A.M on POD 1 or 2 and were started on glucocorticoid replacement with hydrocortisone if cortisol was <15 mg/dL (before January 2021)^32–34^ and <11 mg/dL (January 2021 onwards)^35^ when our protocol was changed due to changes in the cortisol assay.

Prior to initiation of FR, the following criteria had to be met on POD 3: Na level < 145 mEq/L, the patient had to be tolerating fluids by mouth, and the patient could not have evidence of AVP-D (as determined by the inpatient endocrine team). Patients with transient AVP-D before POD 3 were included in the study. If a patient in the FR group developed AVP-D, FR was stopped, and patients were managed as per our institution’s protocol. Patients who developed severe hyponatremia (Na level < 125 mEq/L) or symptoms of hyponatremia regardless of Na level were either directly admitted to the hospital or sent to their local emergency room. Patients with mild hyponatremia (Na level 130–134 mEq/L) were instructed to fluid-restrict to < 1 L per day and patients with moderate hyponatremia (Na level 125–129 mEq/L) were instructed to fluid-restrict to < 800 mL/day. Na levels were then checked daily and patients with moderate hyponatremia were instructed to liberalize fluid intake to 1 L per day once the Na level was >129 mEq/L. FR was stopped once the Na level was >134 mEq/L and the basic metabolic panel was checked 2 days later.

### Endpoints and Assessments

In all patients, Na level was monitored every 8 h in the hospital and on POD 3, 7, 10, and 14 ± 1–2 days if PODs fell on a weekend. Incidence of mild hyponatremia, moderate hyponatremia, severe hyponatremia, hypernatremia, and readmission rates were evaluated. In addition to Na level, the following data were collected: patient demographics, past medical history, body mass index (BMI), tumor characteristics (type, functional status, and size), operative time, intraoperative blood loss, intraoperative fluid balance, operative and postoperative complications, daily fluid intake and output (see below), creatinine, urine specific gravity, patient thirst as indicated below, readmission, and treatment of hyponatremia if it occurred.

All patients received a patient thirst questionnaire that was completed on POD 1, 3, 7, 10, and 14. The intensity of thirst was assessed on a scale of 1–5, with 1 being no thirst, 3 being normal thirst, and 5 being unbearable thirst. Patients were asked to keep track of fluid intake between POD 3 and 14 and mail completed forms to the study team in a prestamped envelope.

### Trial Oversight

The study was approved by our Institutional Review Board (IRB# 201605023), and informed consent was obtained from all patients. An independent data safety monitoring committee was responsible for trial oversight. After 50% of the estimated recruitment had finished the 14-day follow-up of the trial, the committee performed an interim analysis to assess efficacy and futility. External monitoring was also performed to follow protocol adherence, the occurrence of adverse events, and the accuracy of data entry.

### Statistical Analysis

We planned to enroll 300 patients in the initial trial, comparing the moderate FR group to the ad-lib control group. This sample size was calculated to provide 80% power for detecting a 3 mEq/L difference, using a 2-sample *t*-test at a 2-sided alpha level of 0.05, assuming a dropout rate of 25%, based on Na information collected from an earlier clinical trial.^32^ Interim analysis was conducted with 86 (control, *n* = 48; moderate FR, *n* = 38) participants who completed the study before April 2019. An O’Brien–Fleming stopping rule was implemented to control the overall type I error rate at 5%. Conditional power was used to guide futility decisions using a nominal threshold of 25%. Interim analysis yielded an estimated standard deviation of the nadir Na at 5.77 mEq/L, and an estimated treatment difference of 1.5 mEq/L between the control group and moderate FR group. This analysis suggested that continuation of the moderate FR arm (1.8/2 L) would not lead to significant improvement in nadir Na, based on insufficient conditional power (estimated at 23%) at the expected final recruitment. The FR protocol was therefore modified to strict FR to achieve an estimated difference in nadir Na of 2.89 mEq/L between the strict FR and the control group, corresponding to a clinically meaningful effect size of 0.5. Under the assumption of an estimated 50% dropout rate for the strict FR group and 15% dropout rate for the control group, the remaining recruitment of 162 participants (randomized in 2:1 fashion, strict FR, *n* = 108, control, *n* = 54), which combined with the 48 control participants recruited for the interim analysis, would yield 54 strict FR and 94 control patients completing the experiment to ensure that the trial possesses 80% power to detect a 2.98 mEq/L difference, using a 2-sample *t*-test at a 2-sided alpha level of 0.05.

We presented continuous variables as mean (SD), and categorical variables as frequency (proportion in %). Differences in baseline characteristics and clinical outcomes between the control, strict, and moderate FR groups were analyzed using the student’s *t*-test or Fisher’s exact test. In the primary analysis, the Na level between POD 3 and POD 14 was estimated using a linear mixed-effects model. The model included participant-specific effects to address within-participant correlations from repeated assessment. Fixed effects in the model included the assigned randomization group (control, strict FR, and moderate FR), number of days after operation, and operation time. The linear mixed-effect model and its assumptions are detailed in the Supplementary Appendix (Supplementary Methods).

The per-protocol analysis included only patients who adhered to their assigned protocol (strict FR and moderate FR). All patients assigned to the control group were considered compliant by default and thus included in the per-protocol analysis. This decision was made because the control group was designed to reflect population-wide behavior in real-life situations, regardless of personal decisions on fluid intake. In this analysis, participants in the treatment arms were operationally defined as “compliant” if their fluid intake log was available every day between the start of treatment (POD 3) and the end of follow-up (POD 14), and they were compliant to their assigned FR goal for ≥10 days during that period. Sensitivity analyses were conducted to assess the efficacy of strict FR or moderate FR on mean and nadir Na levels using the per-protocol analysis population.

There were no prespecified plans to adjust for multiplicity. Therefore, the results are reported as point estimates and 95% confidence intervals, and confidence intervals should not be used in place of a hypothesis test. All analyses were performed using the R statistical computing environment, version 4.1.3.^36^ All hypothesis tests were 2-sided, and *P* values < .05 were considered to indicate statistical significance.

## Results

### Study Participants

From 2016 through 2023, a total of 300 participants scheduled to undergo resection of a pituitary tumor were screened. After signing consent, 28 patients declined participation for personal reasons, 33 had evidence of AVP-D on POD 3 and were thus excluded, and 44 were excluded for other reasons (eg surgery not performed, critical condition after surgery, or no labs returned).

**Figure 1.**
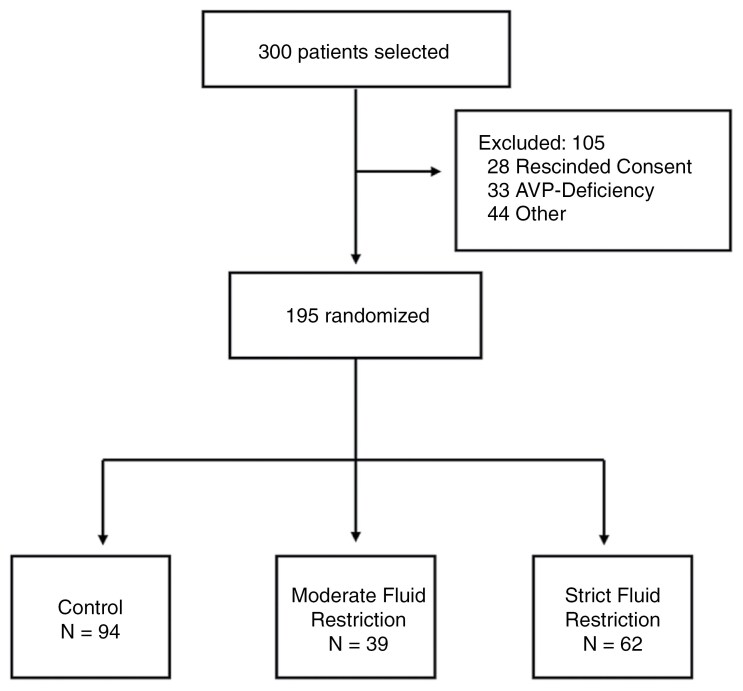
**Randomization and intervention in the intention-to-treat analysis.** Figure showing exclusions for the intention-to-treat analysis population. Participants scheduled for EETS were randomly assigned to the control (*n* = 94) or FR groups (moderate FR = 39, strict FR = 62). All participants were started on postoperative weight-based intravenous fluids until POD 1 and then allowed to drink freely. From POD 3 through POD 14, participants in the moderate FR group were fluid-restricted to 1.8 L per day (2 L per day if weight > 100 kg), and those in the strict FR group were fluid-restricted to 1L per day (1.2 L per day if weight > 100 kg).

Overall, the intention-to-treat analysis population included 195 participants randomly assigned to 1 of 2 FR groups (*n* = 101) POD 3 through POD 14 or to the control group instructed to drink ad-lib (*n* = 94) (Figure 1). Patients were included in the intention-to-treat analysis regardless of whether or not they completed all labs and/or returned fluid logs. In the per-protocol analysis, all control patients were included, and in the FR groups, only patients who sent complete fluid intake logs and adhered to their assigned FR goal ≥ 10 days were included.

Demographics and baseline characteristics in the intention-to-treat analysis were comparable in the 3 groups (Table 1). Among all patients, 52.8% were female, the mean age was 53 years, and the mean BMI was 33.5 kg/m^2^. Most participants had PitNET (95.9%), with 79.0% macroadenomas and 16.9% microadenomas; and among PitNET patients, 65.8% of them were nonfunctioning. A much smaller number of patients had craniopharyngiomas, Rathke’s cleft cysts, and other sellar pathologies (in total, 4.1%). Following EETS, 23 (11.8%) participants were diagnosed with transient AVP-D, defined as being resolved on or before POD 3. No significant difference was seen in the incidence of any other postsurgical complications between groups (Table 2).

**Table 1.** Characteristics of the Patients at Baseline^a^

	Ad-lib	Strict fluid restriction		Moderate fluid restriction		Overall fluid restriction		Overall
Characteristics	(*N* = 94)	(*N* = 62)		(*N* = 39)		(*N* = 101)		(*N* = 195)
			*P* value^b^		*P* value^b^		*P* value^b^	
Demographic characteristics								
Age (year), mean (SD)	55.3 (12.9)	50.1 (16.6)	.04^*^	52.0 (14.5)	.22	50.8 (15.7)	.03^*^	53.0 (14.6)
Sex, *n* (%)			>.99		.71		.89	
Female	49 (52.1%)	32 (51.6%)	–	22 (56.4%)	–	54 (53.5%)	–	103 (52.8%)
Male	45 (47.9%)	30 (48.4%)	–	17 (43.6%)	–	47 (46.5%)	–	92 (47.2%)
Body mass index (BMI), mean (SD)	32.4 (7.14)	34.6 (9.01)	.11	34.3 (7.96)	.21	34.5 (8.58)	.07	33.5 (7.97)
Tumor characteristics					–		–	
Tumor type, *n* (%)			.15		>.99		.69	
Macroadenoma	77 (81.9%)	44 (71.0%)	–	33 (84.6%)	–	77 (76.2%)	–	154 (79.0%)
Microadenoma	15 (16.0%)	12 (19.4%)	–	6 (15.4%)	–	18 (17.8%)	–	33 (16.9%)
Rathke’s cleft cyst	1 (1.1%)	1 (1.6%)	–	0 (0%)	–	1 (1.0%)	–	2 (1.0%)
Craniopharyngioma	0 (0%)	1 (1.6%)	–	0 (0%)	–	1 (1.0%)	–	1 (0.5%)
Other	1 (1.1%)	4 (6.5%)	–	0 (0%)	–	4 (4.0%)	–	5 (2.6%)
Tumor endocrine activity status, *n* (%)			.74		.22		.42	
Nonfunctional	60 (63.8%)	38 (61.3%)	–	25 (64.1%)	–	63 (62.4%)	–	123 (63.1%)
ACTH	22 (23.4%)	15 (24.2%)	–	5 (12.8%)	–	20 (19.8%)	–	42 (21.5%)
GH	6 (6.4%)	8 (12.9%)	–	4 (10.3%)	–	12 (11.9%)	–	18 (9.2%)
TSH	2 (2.1%)	0 (0%)	–	0 (0%)	–	0 (0%)	–	2 (1.0%)
Prolactin	2 (2.1%)	1 (1.6%)	–	4 (10.3%)	–	5 (5.0%)	–	7 (3.6%)
FH	1 (1.1%)	0 (0%)	–	1 (2.6%)	–	1 (1.0%)	–	2 (1.0%)
Mixed	1 (1.1%)	0 (0%)	–	0 (0%)	–	0 (0%)	–	1 (0.5%)
Tumor volume (cm^3^), mean (SD)	4.88 (6.59)	4.73 (5.58)	.94	6.34 (6.34)	.07	5.35 (5.91)	.38	5.12 (6.23)
Prior medical history, *n* (%)			–		–		–	
Arteriosclerosis	13 (13.8%)	0 (0%)	.002^**^	6 (15.4%)	.79	6 (5.9%)	.09	19 (9.7%)
High blood pressure	52 (55.3%)	30 (48.4%)	.42	20 (51.3%)	.71	50 (49.5%)	.47	102 (52.3%)
Asthma	4 (4.3%)	2 (3.2%)	>.99	2 (5.1%)	>.99	4 (4.0%)	>.99	8 (4.1%)
Obesity	34 (36.2%)	32 (51.6%)	.07	8 (20.5%)	.10	40 (39.6%)	.66	74 (37.9%)
Diabetes	18 (19.1%)	15 (24.2%)	.55	6 (15.4%)	.81	21 (20.8%)	.86	39 (20.0%)
Prior endocrine history, *n* (%)								
Primary hypothyroidism	15 (16.0%)	6 (9.7%)	.34	4 (10.3%)	.59	10 (9.9%)	.28	25 (12.8%)
Secondary hypothyroidism	28 (29.8%)	19 (30.6%)	>.99	11 (28.2%)	>.99	30 (29.7%)	>.99	58 (29.7%)
Type 2 diabetes mellitus	16 (17.0%)	13 (21.0%)	.54	7 (17.9%)	>.99	20 (19.8%)	.71	36 (18.5%)
Primary adrenal insufficiency	1 (1.1%)	0 (0%)	>.99	0 (0%)	>.99	0 (0%)	.48	58 (29.7%)
Secondary adrenal insufficiency	14 (14.9%)	13 (21.0%)	.39	5 (12.8%)	>.99	18 (17.8%)	.70	32 (16.4%)
Hypogonadotropic hypogonadism	28 (29.8%)	24 (38.7%)	.30	9 (23.1%)	.53	33 (32.7%)	.76	61 (31.3%)
Other prior endocrine history	6 (6.4%)	7 (11.3%)	.38	0 (0%)	.18	7 (6.9%)	>.99	13 (6.7%)
No prior endocrine history	19 (20.2%)	8 (12.9%)	.28	12 (30.8%)	.26	20 (19.8%)	>.99	39 (20.0%)
Operation characteristics, mean (SD)								
Operative time (min)	249 (87.6)	209 (85.9)	.005^**^	314 (102)	.001^**^	250 (106)	.96	250 (97.0)
Intra-operative blood loss (cc)	158 (200)	118 (102)	.11	241 (231)	.05	166 (175)	.75	162 (187)
Intra-operative fluid balance (cc)	1470 (991)	1390 (753)	.58	1590 (1020)	.52	1470 (867)	.98	1470 (927)
Acute kidney injury, *n* (%)	9 (9.6%)	4 (6.5%)	.57	2 (5.1%)	.51	6 (5.9%)	.42	15 (7.7%)

^a^Mean (SD) or no. patients (%) were reported for each baseline demographic characteristics, tumor characteristics, prior medical history, prior endocrine, history, operation characteristics, postsurgical complications, and readmission characteristics of the 195 patients following pituitary surgery, stratified, by randomization group.

^b^ *P* values were calculated by comparing patients in restriction groups vs ad-lib groups using 2 sample *t*-test or Fisher’s exact test.

^*^ *P* < .1.

^**^ *P* < .05.

^***^ *P* < .001.

**Table 2.** Frequencies and Effect Sizes for Postsurgical Complications Following Intention-To-Treat (ITT) Analysis^a^

	Ad-lib	Strict fluid restriction				Moderate fluid restriction				Overall fluid restriction				Overall
Postsurgical complications	(*N* = 94)	(*N* = 62)				(*N* = 39)				(*N* = 101)				(*N* = 195)
	No. of patients (%)	No. of patients (%)	OR	95% CI	*P* value	No. of patients (%)	OR	95% CI	*P* value	No. of patients (%)	OR	95% CI	*P* value	No. of patients (%)
Meningitis	1 (1.1%)	0 (0%)	0.00	0–93.86	>.99	0 (0%)	0.00	0–36.3	.48	0 (0%)	0.00	0–59.08	>.99	1 (0.5%)
Other infections	0 (0%)	1 (1.6%)	–	–	–	0 (0%)	–	–	–	1 (1.0%)	–	–	–	1 (0.5%)
Cerebrospinal fluid leak	2 (2.1%)	4 (6.5%)	2.47	0.17–35.23	.58	2 (5.1%)	2.89	0.5–30	.28	6 (5.9%)	3.15	0.44–35.82	.22	8 (4.1%)
Cranial nerve deficits	0 (0%)	0 (0%)	–	–	–	0 (0%)	–	–	–	0 (0%)	–	–	–	0 (0%)
Arginine vasopressin deficiency	13 (13.8%)	4 (6.5%)	1.13	0.32–3.53	.79	6 (15.4%)	0.69	0.25–1.8	.51	10 (9.9%)	0.43	0.1–1.49	.19	23 (11.8%)
Intracranial hemorrhage	2 (2.1%)	1 (1.6%)	1.21	0.02–23.87	>.99	1 (2.6%)	0.93	0.07–13.06	>.99	2 (2.0%)	0.76	0.01–14.81	>.99	4 (2.1%)
Other complications	7 (7.4%)	3 (4.8%)	0.67	0.07–3.77	>.99	2 (5.1%)	0.65	0.16–2.47	.56	5 (5.0%)	0.63	0.1–2.92	.74	12 (6.2%)

Abbreviations: OR, odds ratio; 95% CI, 95% confidence interval.

^a^Postsurgical sodium serum level and other conditions were reported for the 195 patients following pituitary surgery, stratified by randomization group. Analyses were conducted following the ITT approach.

^*^ *P* < .1.

^**^ *P* < .05.

^***^ *P* < .001.

### Impact of Fluid Restriction on Na Level

In the intention-to-treat analysis, mean, and nadir Na levels were 139 mEq/L and 135 mEq/L in the control group, 139 mEq/L, and 137 mEq/L in the strict FR group, 139 mEq/L and 136 in the moderate FR group (Table 3). Nadir Na level was significantly higher in the overall FR group (strict + moderate FR) compared with the control group (estimated difference, 1.64 mEq/L; 95% CI, 0.24–3.04; *P* = .02). A similar observation was also found in the subgroup of PitNET patients only, with a higher nadir Na level in the strict FR group when compared with the control group (estimated difference, 2.17 mEq/L; 95% CI, 0.74–3.6; *P* = .003) (Table S1 in the Supplementary Appendix).

**Table 3.** Frequencies and Effect Sizes for Postsurgical Serum Sodium Level Following Intention-To-Treat (ITT) Analysis^a^

	Ad-lib	Strict fluid restriction				Moderate fluid restriction				Overall fluid restriction				Overall
	(*N* = 94)	(*N* = 62)				(*N* = 39)				(*N* = 101)				(*N* = 195)
	Mean (SD)	Mean (SD)	ED	95% CI	*P* value	Mean (SD)	ED	95% CI	*P* value	Mean (SD)	ED	95% CI	*P* value	Mean (SD)
Serum sodium level (mEq/L)^b^														
Nadir^c^	135 (5.56)	137 (3.68)	1.81	0.34–3.27	.02^*^	136 (5.01)	1.38	–0.59–3.34	.17	137 (4.22)	1.64	0.24–3.04	.02^*^	136 (4.97)
Daily average^d^	139 (3.09)	139 (2.56)	0.47	–0.43–1.37	.31	139 (2.60)	0.85	–0.19–1.89	.11	139 (2.57)	0.62	–0.19–1.42	.13	139 (2.84)

Abbreviations: ED, estimated difference; OR, odds ratio; 95% CI, 95% confidence interval; POD, postoperative day.

^a^Postsurgical sodium serum level and kidney condition were reported for the 195 patients following pituitary surgery, stratified by randomization group. Analyses were conducted following ITT approach.

^b^Serum sodium levels were reported by mean (SD). The estimated difference in serum sodium nadir and mean (along with associated 95% CI and *P* value) was calculated using an unpaired *t*-test for each fluid restriction group vs control group.

^c^Serum sodium nadir was defined as the lowest serum sodium level between POD3 and POD 14.

^d^Serum sodium daily average was calculated by taking the daily average of serum sodium level between POD3 and POD14.

^*^ *P* < .1.

^**^ *P* < .05.

^***^ *P* < .001.

**Figure 2.**
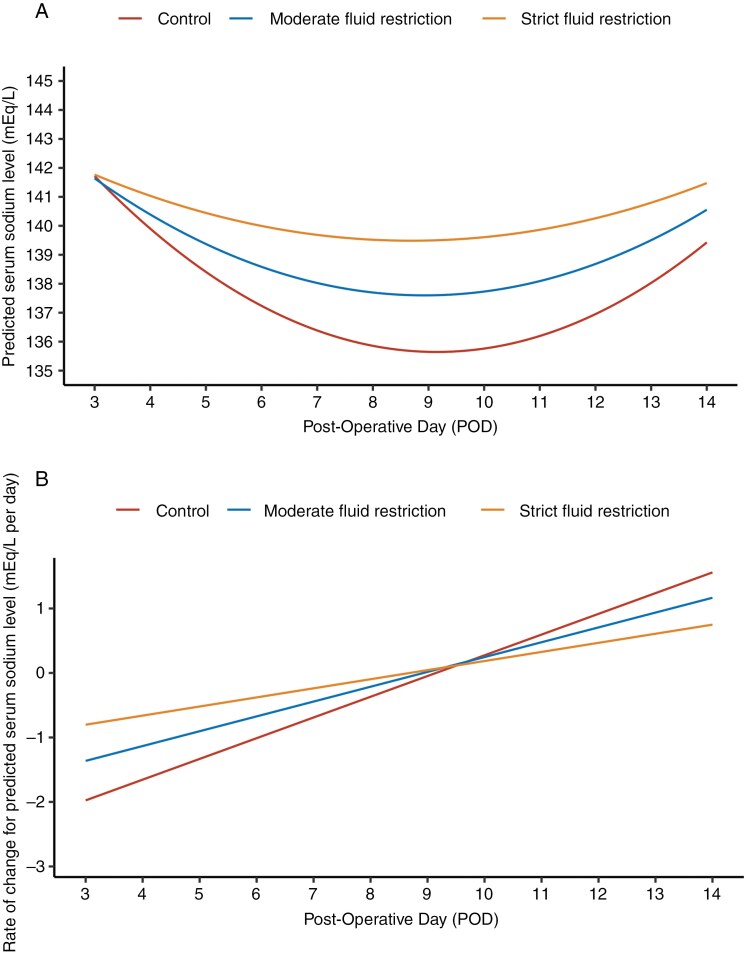
**Temporal dynamics in Na level of patients from POD 3 to POD 14.** Longitudinal trends in estimated Na levels among patients in the control, moderate FR, and strict FR groups from POD 3 to POD 14 are shown. The model was fitted using a linear mixed model, leveraging day-level longitudinal Na-level measurements. Model specification is detailed in Methods S1 in the Supplementary Appendix. **(A)** Temporal trend of estimated Na levels. **(B)** Temporal trend in the rate of change of estimated Na levels.

Using the linear mixed-effects model, we observed a dose–response relation between FR and Na level, such that increasing FR intensity led to a slower decrease in Na level (Figure 2A). After POD 3, the estimated Na level of FR groups, particularly strict FR, is higher than that of the control group. The rate of change (Figure 2B) revealed a similar difference between the groups, with strict FR consistently being more negative than moderate FR and control group (Table S2 in the Supplementary Appendix). FR also led to an earlier occurrence of nadir Na level. In the strict and moderate FR groups, Na level reached a nadir at approximately POD 8.7 and POD 8.9, respectively compared to POD 9.2 for the control group (Figure 2A).

In the per-protocol analysis, FR led to both higher mean and nadir Na levels compared to the control group (Table S3 in the Supplementary Appendix). The overall FR group exhibited a 1.32 mEq/L higher mean Na level (95% CI, 0.37 to 2.27; *P* = .007) and 2.58 mEq/L higher nadir Na level (95% CI, 0.8–4.36; *P* = .005) compared to the control group. In comparisons of strict FR versus the control group, mean Na increased further by 1.52 mEq/L (95% CI, 0.37–2.66; *P* = .01), and nadir Na level increased by 3.50 mEq/L (95% CI, 1.68 to 5.31; *P* < .001).

### Impact of Fluid Restriction on Incidence of Hyponatremia and Readmission

The incidence of mild, moderate, and severe hyponatremia in the intention-to-treat population was 16.1%, 4.8%, and 0% for the strict FR group, 17.9%, 5.1%, and 5.1% for the moderate FR group, and 19.1%, 5.3%, and 7.4% for the control group (Table 4). Importantly, the odds of having severe hyponatremia were significantly lower in the strict FR (0%) vs. the control group (7.4%) (95% CI, 0.00–1.02, *P* = .04). In addition, the odds of having hyponatremia of any severity (nadir Na level < 135 mEq/L) in the overall FR group (10.3%) was also significantly lower when compared to the control group (31.9%) in the per-protocol analysis (95% CI, 0.04–0.91; *P* = .03) (Table S3 in the Supplementary Appendix).

**Table 4.** Frequencies and Effect Sizes for Postsurgical Hyponatremia and Readmission Status Following Intention-To-Treat (ITT) Analysis^a^

	Ad-lib	Strict fluid restriction				Moderate fluid restriction				Overall fluid restriction				Overall
	(*N* = 94)	(*N* = 62)				(*N* = 39)				(*N* = 101)				(*N* = 195)
	No. of patients (%)	No. of patients (%)	OR	95% CI	*P* value	No. of patients (%)	OR	95% CI	*P* value	No. of patients (%*)*	OR	95% CI	*P* value	No. of patients (%)
Hyponatremia^b^														
Any severity	30 (31.9%)	13 (21.0%)	0.57	0.24–1.26	.15	11 (28.2%)	0.84	0.33–2.03	.84	24 (23.8%)	0.67	0.34–1.31	.26	54 (27.7%)
Mild	18 (19.1%)	10 (16.1%)	0.81	0.31–2.03	.68	7 (17.9%)	0.92	0.3–2.61	>.99	17 (16.8%)	0.86	0.38–1.90	.71	35 (17.9%)
Moderate	5 (5.3%)	3 (4.8%)	0.91	0.14–4.86	>.99	2 (5.1%)	0.96	0.09–6.21	>.99	5 (5.0%)	0.93	0.21–4.18	>.99	10 (5.1%)
Severe	7 (7.4%)	0 (0%)	0.00	0–1.02	.04^*^	2 (5.1%)	0.67	0.07–3.77	>.99	2 (2.0%)	0.25	0.02–1.37	.09	35 (17.9%)
Readmission														
All reasons	14 (14.9%)	4 (6.5%)	0.39	0.09–1.32	.13	8 (20.5%)	1.48	0.49–4.25	.45	12 (11.9%)	0.76	0.30–1.89	.53	26 (13.3%)
Due to Hyponatremia	6 (6.4%)	1 (1.6%)	0.46	0.01–7.63	>.99	3 (7.7%)	0.81	0.09–6.38	>.99	4 (4.0%)	0.68	0.10–4.26	.70	10 (5.1%)

Abbreviations: ED, estimated difference; OR, odds ratio; 95% CI, 95% confidence interval; POD, postoperative day.

^a^Postsurgical hyponatremia and readmission status were reported for the 195 patients following pituitary surgery, stratified by randomization group. Analyses were conducted following the ITT approach. Postsurgical hyponatremia and readmissions were reported by no. of patients (%). The odds ratio of each condition (along with the associated 96% CI and *P* value) was calculated using Fisher’s exact test for each fluid restriction group vs the control group.

^b^Hyponatremia was defined as serum sodium concentration < 135mEq/L. Moderate hyponatremia: 125–129mEq/L; Severe hyponatremia: <125 mEq/L.

^*^ *P* < .1.

^**^ *P* < .05.

^***^ *P* < .001.

In the intention-to-treat analysis, we observed a lower rate of readmission due to hyponatremia among both strict FR (1.6%) and overall FR group (4.0%) compared to the control group (6.4%) (Table 4). These findings were also observed in the per-protocol population, with both strict FR (0%) and overall FR group (3.4%) having a lower rate of readmission due to hyponatremia when compared to the control group (6.4%) (Table S3 in the Supplementary Appendix).

### Treatment Compliance and Thirst

A total of 29 patients in the FR groups (15 (24.2%) strict FR and 14 (48.7%) moderate FR) returned complete fluid intake logs and were compliant with FR. The mean daily fluid intake between POD 3 and 14 was 1097 mL for the strict FR group, 1405 mL for the moderate FR group, and 1740 mL for the control group. Comparing intervention arms, participants in the strict FR group were compliant with their assigned protocol for significantly fewer days (7.4 days) than those in the moderate FR group (10.0 days) (95% CI, 0.34–4.80; *P* = .03). Participants in both strict (estimated difference, –633.0 mL/day; 95% CI, –898.7 to –387.3; *P* < .01) and moderate FR groups (estimated difference, –335.2 mL/day; 95% CI, –629.1 to –41.3; *P* = .03) drank less fluid throughout the postoperative follow-up period compared to participants in the control group (Figure S1 in Supplementary Appendix). Although mean thirst scores were similar among the 3 randomized groups (strict FR: 3.16; moderate FR: 2.89; control: 2.99; *P* = .37), individual thirst score was found to be a significant predictor for overall compliance to the assigned FR protocol, adjusting for sex, BMI, AVP-D, and age (odds ratio (OR), 0.12; 95% CI, 0.02–0.70; *P* = .02) (Figure S2 in the Supplementary Appendix), suggesting individual heterogeneity among participants in terms of thirst. In addition, males had lower compliance to FR compared to females (OR, 0.1; 95% CI, 0.02–0.57; *P* = .009).

## Discussion

Delayed hyponatremia is the most common complication after EETS and the most common cause of 30-day readmission (up to 60%).^4,12,37–39^ Reported rates of delayed hyponatremia range between 0 and 39%,^3,5,6,24,40^ and lead to readmission within the first 30 days in 4–5%^4,5,9,30,31^ of patient cohorts drinking ad-lib. Results of our randomized controlled study definitively show a causal relationship between postoperative fluid management and delayed hyponatremia and confirm the results of published retrospective and single-arm prospective studies suggesting a decrease in the incidence of delayed hyponatremia and readmission with the implementation of a postoperative FR protocol.^3,5,8,24,28,30,31^ The mechanism of delayed hyponatremia in the post-EETS setting is thought to be SIADH, but whether this is due to aberrant release of AVP due to injury of the posterior pituitary gland or stalk or due to an altered homeostatic set point for AVP release remains to be determined.

The most significant finding in our study is that the incidence of developing severe hyponatremia was significantly lower in the strict FR group compared to the control group (0% vs. 7.4%; 95% CI, 0.00–1.02; *P* = .04) in the intention-to-treat population, and the odds of developing hyponatremia of any severity was significantly lower in the overall FR group (strict and moderate) compared to the control group in the per-protocol analysis, which included only patients in the intervention groups who returned complete fluid logs and recorded data for at least 10 days (10.3% vs. 31.9%, 95% CI, 0.04–0.91; *P* = .03). Furthermore, the rate of readmission due to hyponatremia was decreased in the strict FR group (1.6%) and overall FR group (4%) compared to the control group (6.4%) in the intention-to-treat analysis and decreased in both the strict FR (0%) and overall FR group (3.4%) compared to the control group (6.4%) in the per-protocol analysis. Although our study was not powered to investigate rates of readmission, our results are consistent with other studies demonstrating a lower incidence of readmission for hyponatremia with postoperative fluid restriction.^24,27–29^ Unlike patients in previous studies,^28,29^ in which Na level was checked once after surgery, patients in our study had Na level checked 4 times after POD 3, which could have led to earlier recognition and management of delayed hyponatremia in the outpatient setting, thus contributing to lower readmission rates in all groups in the current study.

Although routine postoperative FR is a safe and effective way to prevent delayed hyponatremia, the duration and amount of FR and timing of postoperative Na level monitoring remain to be elucidated. Ideal protocols would minimize the number of laboratories draws and the severity of FR in patients. In our study, nadir Na level was higher in the overall FR group compared to the control group (estimated difference, 1.64 mEq/L; 95% CI, 0.24–3.04; *P* = .02) and occurred between POD 8 and 9, similar to other studies.^8,26,27^ In addition, the rate of Na level decrease was slower in both FR groups compared to the control group, and Na level began to trend up in all groups by POD 10 suggesting that the optimal duration of FR may be between POD 3 and 10. Furthermore, based on our data, more streamlined monitoring of Na levels in fluid-restricted patients may be sufficient. Although this will require further validation, limited Na level checks around POD 8 appear reasonable in these patients. Patients not discharged on FR and/or who develop hyponatremia need more frequent monitoring.

An additional important finding of our study is that FR is safe in patients who develop transient AVP-D after EETS and in patients with pituitary pathology other than PitNET. Few studies evaluating postoperative FR have included patients with transient AVP-D after surgery. Several FR studies excluded all patients with AVP-D or did not specifically mention whether patients with transient AVP-D were excluded.^24,26,28–30^ In our study, 10 patients (9.9%) randomized to either of the 2 FR arms had evidence of transient postoperative AVP-D and did not develop hypernatremia or AVP-D throughout the study. Similar to other retrospective studies,^24,26,28,30^ we found that FR is safe in patients with non-PitNET sellar lesions such as Rathke’s cleft cysts and craniopharyngiomas.

The prospective design of our study enabled us to capture data on fluid intake and compliance. Based on fluid intake logs, patients drank on average 1097 mL, 1405 mL, and 1740 mL/day in the strict FR, moderate FR, and control groups, respectively. Overall, participants in both FR groups had consistently reduced fluid intake compared to the control group, although more patients in the moderate FR group were compliant with FR compared to patients in the strict FR group. Although this will require further validation, our results suggest that restricting patients to <1.1 to 1.4 L per day may be a realistic and effective fluid target for patients. Interestingly, despite the mean thirst score being similar among all 3 groups, we found that an individual’s thirst score was a significant predictor of overall compliance with the participant’s assigned fluid restriction protocol. In addition, we found that the female gender was a predictor of compliance.

Limitations to our study include a relatively large drop-out rate prior to randomization due to patient preference and a diagnosis of AVP-D on POD 3, some of which were likely transient. In addition, patient compliance with filling out and returning fluid logs after discharge may have introduced selection bias since patients who returned fluid logs may have been most likely to comply with the FR. However, analysis of patients in the intention-to-treat and the per-protocol analysis population both showed lower rates of hyponatremia and readmission with FR. Although we did not evaluate postoperative hormone deficiencies, all patients in our study were managed with the same perioperative protocol to assess the HPA axis so we do not believe that there were patients with untreated adrenal insufficiency. Furthermore, we have previously shown that rates of hyponatremia are not impacted by the use of perioperative steroids,^32^ and inadequate thyroid and/or glucocorticoid replacement was not found to contribute to delayed hyponatremia in another study.^29^

In conclusion, delayed hyponatremia after EETS remains a common issue. To our knowledge, this is the first randomized prospective controlled trial to demonstrate that FR is an effective and low-risk method of preventing delayed hyponatremia. We also demonstrate that postoperative FR is safe in patients who develop transient AVP-D before POD 3 and who have pituitary adenoma as well as other pituitary lesions, such as craniopharyngiomas or Rathke’s cleft cysts. The heterogeneity of outcomes in studies of FR is likely due to varying duration (4–14 days) and amount (1–2.5 L per day) of FR.^11^ Further studies are needed to determine the optimal duration and daily volume of FR needed to elicit a clinically significant decrease in the rate of delayed hyponatremia and readmissions. The analysis of patient compliance in our FR protocol adds a novel dimension to our study and also raises the intriguing question of what methodologies might improve patient compliance to FR and other similar patient-driven interventions, which can be addressed in future studies.

## Supplementary material

Supplementary material is available online at *Neuro-Oncology* (https://academic.oup.com/neuro-oncology).

noaf069_suppl_Supplementary_Materials

## Data Availability

Statement declaring that the data will be made available upon reasonable request.

## References

[1] Ostrom QT, Price M, Neff C, et al CBTRUS statistical report: primary brain and other central nervous system tumors diagnosed in the United States in 2016–2020. Neuro-Oncology. 2023;25(12 Suppl 2):iv1–iv99. 10.1093/neuonc/noad149 PMC10550277 37793125

[2] Ezzat S, Asa SL, Couldwell WT, et al The prevalence of pituitary adenomas. Cancer. 2004;101(3):613–619. 15274075 10.1002/cncr.20412

[3] Yu S, Taghvaei M, Reyes M, et al Delayed symptomatic hyponatremia in transsphenoidal surgery: systematic review and meta-analysis of its incidence and prevention with water restriction. Clin Neurol Neurosurg. 2022;214:107166. 35158166 10.1016/j.clineuro.2022.107166

[4] Bohl MA, Ahmad S, Jahnke H, et al Delayed hyponatremia is the most common cause of 30-day unplanned readmission after transsphenoidal surgery for pituitary tumors. Neurosurgery. 2016;78(1):84–90. 26348011 10.1227/NEU.0000000000001003

[5] Perez-Vega C, Tripathi S, Domingo RA, et al Fluid restriction after transsphenoidal surgery for the prevention of delayed hyponatremia: a systematic review and meta-analysis. Endocr Pract. 2021;27(9):966–972. 34265453 10.1016/j.eprac.2021.07.003

[6] Hong YG, Kim SH, Kim EH. Delayed hyponatremia after transsphenoidal surgery for pituitary adenomas: a single institutional experience. Brain Tumor Res Treat. 2021;9(1):16–20. 33913267 10.14791/btrt.2021.9.e5 PMC8082282

[7] Hussein Z, Tzoulis P, Marcus HJ, et al The management and outcome of hyponatraemia following transsphenoidal surgery: a retrospective observational study. Acta Neurochir (Wien). 2022;164(4):1135–1144. 35079890 10.1007/s00701-022-05134-9 PMC8967808

[8] Takeuchi K, Nagatani T, Okumura E, Wakabayashi T. A novel method for managing water and electrolyte balance after transsphenoidal surgery: preliminary study of moderate water intake restriction. Nagoya J Med Sci. 2014;76(1–2):73–82. 25129993 PMC4345719

[9] Younus I, Gerges MM, Dobri GA, Ramakrishna R, Schwartz TH. Readmission after endoscopic transsphenoidal pituitary surgery: analysis of 584 consecutive cases. J Neurosurg. 2019;133(4):1242–1247. 31561225 10.3171/2019.7.JNS191558

[10] Halawa I, Andersson T, Tomson T. Hyponatremia and risk of seizures: a retrospective cross-sectional study. Epilepsia. 2011;52(2):410–413. 21314679 10.1111/j.1528-1167.2010.02939.x

[11] Koul PA, Khan UH, Jan RA, et al Osmotic demyelination syndrome following slow correction of hyponatremia: possible role of hypokalemia. Indian J Crit Care Med. 2013;17(4):231–233. 24133331 10.4103/0972-5229.118433 PMC3796902

[12] Cote DJ, Alzarea A, Acosta MA, et al Predictors and rates of delayed symptomatic hyponatremia after transsphenoidal surgery: a systematic review [corrected]. World Neurosurg. 2016;88:1–6. 26805685 10.1016/j.wneu.2016.01.022

[13] Sata A, Hizuka N, Kawamata T, Hori T, Takano K. Hyponatremia after transsphenoidal surgery for hypothalamo-pituitary tumors. Neuroendocrinology. 2006;83(2):117–122. 16864995 10.1159/000094725

[14] Olson BR, Rubino D, Gumowski J, Oldfield EH. Isolated hyponatremia after transsphenoidal pituitary surgery. J Clin Endocrinol Metab. 1995;80(1):85–91. 7829644 10.1210/jcem.80.1.7829644

[15] Hannon MJ, Thompson CJ. The syndrome of inappropriate antidiuretic hormone: prevalence, causes and consequences. Eur J Endocrinol. 2010;162(Suppl 1):S5–12. 20164214 10.1530/EJE-09-1063

[16] Jessani N, Jehangir W, Behman D, Yousif A, Spiler IJ. Secondary adrenal insufficiency: an overlooked cause of hyponatremia. J Clin Med Res. 2015;7(4):286–288. 25699130 10.14740/jocmr2041w PMC4330026

[17] Whitaker SJ, Meanock CI, Turner GF, et al Fluid balance and secretion of antidiuretic hormone following transsphenoidal pituitary surgery. A preliminary series. J Neurosurg. 1985;63(3):404–412. 4020468 10.3171/jns.1985.63.3.0404

[18] Kröll M, Juhler M, Lindholm J. Hyponatraemia in acute brain disease. J Intern Med. 1992;232(4):291–297. 1328460 10.1111/j.1365-2796.1992.tb00588.x

[19] Gupta M, Singh S, Chauhan B. Comparative study of complete nasal packing with and without airways. B-Ent. 2011;7(2):91–96. 21838092

[20] Verbalis JG, Goldsmith SR, Greenberg A, et al Diagnosis, evaluation, and treatment of hyponatremia: expert panel recommendations. Am J Med. 2013;126(10 Suppl 1):S1–42. 10.1016/j.amjmed.2013.07.006 24074529

[21] Yuen KCJ, Ajmal A, Correa R, Little AS. Sodium perturbations after pituitary surgery. Neurosurg Clin N Am. 2019;30(4):515–524. 31471059 10.1016/j.nec.2019.05.011

[22] Ayus JC, Olivero JJ, Frommer JP. Rapid correction of severe hyponatremia with intravenous hypertonic saline solution. Am J Med. 1982;72(1):43–48. 7058821 10.1016/0002-9343(82)90575-7

[23] Indirli R, Ferreira de Carvalho J, Cremaschi A, et al Tolvaptan in the management of acute euvolemic hyponatremia after transsphenoidal surgery: a retrospective single-center analysis. Front Endocrinol (Lausanne). 2021;12:689887. 34108941 10.3389/fendo.2021.689887 PMC8181462

[24] Winograd D, Staggers KA, Sebastian S, et al An effective and practical fluid restriction protocol to decrease the risk of hyponatremia and readmissions after transsphenoidal surgery. Neurosurgery. 2020;87(4):761–769. 31993647 10.1093/neuros/nyz555

[25] Bohl MA, Ahmad S, White WL, Little AS. Implementation of a postoperative outpatient care pathway for delayed hyponatremia following transsphenoidal surgery. Neurosurgery. 2018;82(1):110–117. 28449052 10.1093/neuros/nyx151

[26] Matsuyama J, Ikeda H, Sato S, et al Early water intake restriction to prevent inappropriate antidiuretic hormone secretion following transsphenoidal surgery: low BMI predicts postoperative SIADH. Eur J Endocrinol. 2014;171(6):711–716. 25227132 10.1530/EJE-14-0530

[27] Snyder MH, Asuzu DT, Shaver DE, Vance ML, Jane JA. Routine postoperative fluid restriction to prevent syndrome of inappropriate antidiuretic hormone secretion after transsphenoidal resection of pituitary adenoma. J Neurosurg. 2022;136(2):405–412. 34330096 10.3171/2021.1.JNS203579

[28] Burke WT, Cote DJ, Iuliano SI, Zaidi HA, Laws ER. A practical method for prevention of readmission for symptomatic hyponatremia following transsphenoidal surgery. Pituitary. 2018;21(1):25–31. 29075986 10.1007/s11102-017-0843-5

[29] Deaver KE, Catel CP, Lillehei KO, Wierman ME, Kerr JM. Strategies to reduce readmissions for hyponatremia after transsphenoidal surgery for pituitary adenomas. Endocrine. 2018;62(2):333–339. 29961198 10.1007/s12020-018-1656-7

[30] Cooper O, Lis R, Bonert V, et al Fluid restriction reduces delayed hyponatremia and hospital readmissions after transsphenoidal surgery. J Clin Endocrinol Metab. 2023;108(8):e623–e633. 36723998 10.1210/clinem/dgad066

[31] Castle-Kirszbaum M, Goldschlager T, Shi MDY, Kam J, Fuller PJ. Postoperative fluid restriction to prevent hyponatremia after transsphenoidal pituitary surgery: an updated meta-analysis and critique. J Clin Neurosci. 2022;106:180–184. 36369079 10.1016/j.jocn.2022.10.032

[32] Sterl K, Thompson B, Goss CW, et al Withholding perioperative steroids in patients undergoing transsphenoidal resection for pituitary disease: randomized prospective clinical trial to assess safety. Neurosurgery. 2019;85(2):E226–E232. 30325449 10.1093/neuros/nyy479

[33] Marko NF, Gonugunta VA, Hamrahian AH, et al Use of morning serum cortisol level after transsphenoidal resection of pituitary adenoma to predict the need for long-term glucocorticoid supplementation. J Neurosurg. 2009;111(3):540–544. 19326985 10.3171/2008.12.JNS081265

[34] Marko NF, Hamrahian AH, Weil RJ. Immediate postoperative cortisol levels accurately predict postoperative hypothalamic–pituitary–adrenal axis function after transsphenoidal surgery for pituitary tumors. Pituitary. 2010;13(3):249–255.

[35] Javorsky BR, Raff H, Carroll TB, et al New cutoffs for the biochemical diagnosis of adrenal insufficiency after ACTH stimulation using specific cortisol assays. J Endocr Soc. 2021;5(4):bvab022. 33768189 10.1210/jendso/bvab022 PMC7975762

[36] R Core Team. R: A Language and Environment for Statistical Computing [Computer Program]. Vienna, Austria, 2023.

[37] Kristof RA, Rother M, Neuloh G, Klingmüller D. Incidence, clinical manifestations, and course of water and electrolyte metabolism disturbances following transsphenoidal pituitary adenoma surgery: a prospective observational study. J Neurosurg. 2009;111(3):555–562. 19199508 10.3171/2008.9.JNS08191

[38] Krogh J, Kistorp CN, Jafar-Mohammadi B, et al Transsphenoidal surgery for pituitary tumours: frequency and predictors of delayed hyponatraemia and their relationship to early readmission. Eur J Endocrinol. 2018;178(3):247–253. 29263154 10.1530/EJE-17-0879

[39] Cote DJ, Dasenbrock HH, Muskens IS, et al Readmission and other adverse events after transsphenoidal surgery: prevalence, timing, and predictive factors. J Am Coll Surg. 2017;224(5):971–979. 28279778 10.1016/j.jamcollsurg.2017.02.015

[40] Bennett BL, Hew-Butler T, Rosner MH, Myers T, Lipman GS. Wilderness medical society clinical practice guidelines for the management of exercise-associated hyponatremia: 2019 update. Wilderness Environ Med. 2020;31(1):50–62. 32044213 10.1016/j.wem.2019.11.003

