# Supplementary material for: Postoperative fluid restriction to prevent delayed hyponatremia after endoscopic transsphenoidal surgery
Source: Neuro Oncol. 2025 Mar 14;27(7):1746–57. doi: 10.1093/neuonc/noaf069 (PMC12417837; doi:10.1093/neuonc/noaf069)
Supplement: noaf069_suppl_Supplementary_Materials [file noaf069_suppl_supplementary_materials.docx]

**Supplementary Appendix**

This appendix has been provided by the authors to give readers additional information about their work.

Supplement to: Doriann Klassen, Shinghei Mok et al Post-operative Fluid Restriction to Prevent Delayed Hyponatremia

**POST-OPERATIVE FLUID RESTRICTION TO PREVENT DELAYED HYPONATREMIA**

**SUPPLEMENTARY MATERIALS**

**TABLE OF CONTENTS PAGE**

1. **SUPPLEMENTARY METHODS**

Method S1. Linear Mixed Model Specification………………………………….………..…………………………...3-4

1. **SUPPLEMENTARY TABLES**

Table S1. Frequencies and Effect Sizes for Post-Surgical Serum Sodium Level and Other Conditions among Pituitary Adenoma Patients Following Intention-To-Treat (ITT) Analysis.………………..…………...5

Table S2. Estimated Sodium Level and Rate of Change Derived from Linear Mixed Model…………….…………6

Table S3. Frequencies and Effect Sizes for Post-surgical Serum Sodium Level and Other Conditions Following Per-Protocol Analysis……………………………………………………………………………………………………...7

1. **SUPPLEMENTARY FIGURES**

Figure legend……………………………………………………………………………………………………………….8

Figure S1. Fluid Intake of Patients from POD 3 to POD 14……………………………………………………..…….9

Figure S2. Odds Ratio for Compliance to Fluid Restriction Protocol………………..………………………………10

**Methods S1. Linear Mixed Model Specification**

We modeled serum sodium (Na) levels between post-operative day (POD) 3 and POD 14 using a linear mixed model (LMM) with subject-level random effects for slope across time. We denoted the Na level of $i$-th participant at the $j$-th measurement as $Y_{ij}$, the vector of fixed variables as $X_{i}$, and the time-varying variables as $Z_{i}\left( t \right)$.

$$Y_{ij}=X_{i}\gamma+Z_{i}\left( t_{j} \right)\beta_{i}^{(g)}+\varepsilon_{ij}, \beta_{i}^{(g)}\sim N\left( \beta^{(g)},\sigma_{\beta}^{2} \right),\epsilon_{ij}\sim N(0,\sigma_{\epsilon}^{2})$$

$\gamma$ and $\beta$ represented the fixed effects of baseline characteristics (protocol group assignment, operation time), and time-varying variable (POD). Depending on the group assignment (g = ad-lib, moderate, or for strict), the fixed effects of time-varying variables $\beta^{(g)}$are different. The random effects $\beta_{i}^{(g)}$allows the effects of time-varying variables to be different between patients in the same group. $\varepsilon_{ij}$ indicated the random error for the $i$-th participant at the $j$-th measurement, following a 𝑁(0, $\sigma_{\epsilon}^{2}$) distribution. We used the package lme4 to fit the above LMM model.

The time-varying term in the above model formula was represented using a quadratic spline, i.e. $Z_{i}\left( t \right)=(t-3,\frac{\left( t-3 \right)^{2}}{2}$),

$$Z_{i}(t)\beta^{\left( g \right)}={\beta_{1}}^{\left( g \right)}\left( t-3 \right)+\frac{{\beta_{2}}^{\left( g \right)}}{2}\left( t-3 \right)^{2}+C$$

$$=\frac{{\beta_{2}}^{\left( g \right)}}{2}{(t-3+\frac{{\beta_{1}}^{\left( g \right)}}{{\beta_{2}}^{\left( g \right)}})}^{2}-{\frac{{\beta_{2}}^{\left( g \right)}}{2}\left( 3-\frac{{\beta_{1}}^{\left( g \right)}}{{\beta_{2}}^{\left( g \right)}} \right)}^{2}+C, \beta^{\left( g \right)}=(\beta_{1}^{\left( g \right)},\beta_{2}^{\left( g \right)})$$

1. The estimated rate of change of Na level on POD 3 ($t=3)$ is

$$\frac{d}{dx}{(Z}_{i}(t)\beta^{\left( g \right)})={\beta_{1}}^{\left( g \right)}+{\beta_{2}}^{\left( g \right)}\left( t-3 \right)|_{t=3}={\beta_{1}}^{\left( g \right)}$$

The difference in the rate of change of Na level between the strict FR group and the control group is

$${\beta_{1}}^{strict}-{\beta_{1}}^{ad-lib}$$

and the difference between the moderate FR group and the control group is ${\beta_{1}}^{moderate}-{\beta_{1}}^{ad-lib}$

1. The estimated POD at the nadir Na level is

$$t=3-\frac{{\beta_{1}}^{\left( g \right)}}{{\beta_{2}}^{\left( g \right)}}$$

1. The estimated nadir Na level is

$$-{\frac{{\beta_{2}}^{\left( g \right)}}{2}\left( 3-\frac{{\beta_{1}}^{\left( g \right)}}{{\beta_{2}}^{\left( g \right)}} \right)}^{2}+C$$

The difference in nadir Na level between the strict FR group and the control group is

$${\frac{{\beta_{2}}^{ad-lib}}{2}\left( 3-\frac{{\beta_{1}}^{ad-lib}}{{\beta_{2}}^{ad-lib}} \right)}^{2}-{\frac{{\beta_{2}}^{strict}}{2}\left( 3-\frac{{\beta_{1}}^{strict}}{{\beta_{2}}^{strct}} \right)}^{2}$$

and the difference between the moderate FR group and the control group is

$${\frac{{\beta_{2}}^{ad-lib}}{2}\left( 3-\frac{{\beta_{1}}^{ad-lib}}{{\beta_{2}}^{ad-lib}} \right)}^{2}-{\frac{{\beta_{2}}^{moderate}}{2}\left( 3-\frac{{\beta_{1}}^{moderate}}{{\beta_{2}}^{moderate}} \right)}^{2}$$

**Table S1. Frequencies and Effect Sizes for Post-Surgical Serum Sodium Level and Other Conditions among PitNet Patients Following Intention-To-Treat (ITT) Analysis.^a^**

|  | **Control** | **Strict Fluid Restriction** | | | | **Moderate Fluid Restriction** | | | | **Overall Fluid Restriction** | | | | **Overall** |
| --- | --- | --- | --- | --- | --- | --- | --- | --- | --- | --- | --- | --- | --- | --- |
|  | **(N=92)** | **(N=56)** | | | | **(N=39)** | | | | **(N=95)** | | | | **(N=187)** |
|  | *mean (SD)* | *mean (SD)* | *ED* | *95% CI* | *P value* | *mean (SD)* | *ED* | *95% CI* | *P value* | *mean (SD)* | *ED* | *95% CI* | *P value* | *mean (SD)* |
| **Serum sodium level (mEq/L)^b^** |  |  |  |  |  |  |  |  |  |  |  |  |  |  |
| Nadir^c^ | 135 (5.43) | 137 (3.37) | 2.17 | 0.74 — 3.6 | 0.003** | 136 (5.01) | 1.24 | -0.72 — 3.19 | 0.21 | 137 (4.12) | 1.79 | 0.39 — 3.18 | 0.01* | 136 (4.88) |
| Daily average^d^ | 139 (3.02) | 139 (2.49) | 0.67 | -0.23 — 1.58 | 0.15 | 139 (2.60) | 0.77 | -0.27 — 1.81 | 0.14 | 139 (2.52) | 0.71 | -0.09 — 1.52 | 0.08 | 139 (2.79) |
|  | *no. of patients*  *(%)* | *no. of patients (%)* | *OR* | *95% CI* | *P value* | *no. of patients*  *(%)* | *OR* | *95% CI* | *P value* | *no. of patients*  *(%)* | *OR* | *95% CI* | *P value* | *no. of patients*  *(%)* |
| **Post-surgical conditions^e^** |  |  |  |  |  |  |  |  |  |  |  |  |  |  |
| Hyponatremia^f^ |  |  |  |  |  |  |  |  |  |  |  |  |  |  |
| Any severity | 29 (31.5%) | 8 (14.3%) | 0.54 | 0.26 — 1.11 | 0.09 | 11 (28.2%) | 0.36 | 0.13 — 0.91 | 0.02* | 19 (20.0%) | 0.85 | 0.34 — 2.07 | 0.84 | 48 (25.7%) |
| Mild | 18 (19.6%) | 6 (10.7%) | 0.65 | 0.27 — 1.52 | 0.33 | 7 (17.9%) | 0.50 | 0.15 — 1.42 | 0.18 | 13 (13.7%) | 0.90 | 0.29 — 2.54 | >0.99 | 31 (16.6%) |
| Moderate | 5 (5.4%) | 2 (3.6%) | 0.77 | 0.15 — 3.69 | 0.74 | 2 (5.1%) | 0.65 | 0.06 — 4.12 | 0.71 | 4 (4.2%) | 0.94 | 0.09 — 6.07 | >0.99 | 9 (4.8%) |
| Severe | 6 (6.5%) | 0 (0%) | 0.31 | 0.03 — 1.79 | 0.16 | 2 (5.1%) | 0.00 | 0 — 1.36 | 0.08 | 2 (2.1%) | 0.78 | 0.07 — 4.61 | >0.99 | 31 (16.6%) |
| **Readmission** |  |  |  |  |  |  |  |  |  |  |  |  |  |  |
| All reasons | 13 (14.1%) | 3 (5.4%) | 0.79 | 0.3 — 2.03 | 0.66 | 8 (20.5%) | 0.34 | 0.06 — 1.31 | 0.11 | 11 (11.6%) | 1.57 | 0.51 — 4.60 | 0.43 | 24 (12.8%) |
| Due to Hyponatremia | 5 (5.4%) | 0 (0%) | 0.61 | 0.07 — 4.51 | 0.68 | 3 (7.7%) | 0.00 | 0 — 5.47 | 0.51 | 3 (3.2%) | 0.96 | 0.10 — 8.05 | >0.99 | 8 (4.3%) |
| *** P<.001, **P<.05, *P<.1 | | | | | | | | | | | | | | |
| Abbreviations: ED, estimated difference; OR, odds ratio; 95% CI, 95% confidence interval; POD, postoperative day | | | | | | | | | | | | | | |
| ^a^: Post-surgical sodium serum level and other conditions were reported for the 195 patients following pituitary surgery, stratified by randomization group. Analyses were conducted following ITT approach. | | | | | | | | | | | | | | |
| ^b^: Serum sodium level were reported by mean (SD). Estimated difference in serum sodium nadir and mean (along with associated 95% CI and P value) was calculated using unpaird T test for each fluid restriction group vs control group. | | | | | | | | | | | | | | |
| ^c^: Serum sodium nadir was defined as the lowest serum sodium level between POD3 and POD 14. | | | | | | | | | | | | | | |
| ^d^: Serum sodium daily average was calculated from taking the daily average of serum sodium level between POD3 to POD14. | | | | | | | | | | | | | | |
| ^e^: Post-surgical conditions were reported by no. of patients (%). Odds ratio of each condition (along with associated 96% CI and P value) was calculated using Fisher's exact test for each fluid restriction group vs control group. | | | | | | | | | | | | | | |
| ^f^: Hyponatremia was defined as serum sodium concentration <135mEq/L. Moderate hyponatremia: 125-129mEq/L; Severe hyponatremia: <125mEq/L. | | | | | | | | | | | | | | |

**Table S2. Estimated Sodium Level and Rate of Change Derived from Linear Mixed Model**

|  | **Ad-lib** | | **Strict Fluid Restriction** | | **Moderate Fluid Restriction** | |
| --- | --- | --- | --- | --- | --- | --- |
| POD | estimated sodium level (mEq/L)^a^ | estimated rate of change (mEq/L)^b^ | estimated sodium level (mEq/L)^a^ | estimated rate of change (mEq/L)^b^ | estimated sodium level (mEq/L)^a^ | estimated rate of change (mEq/L)^b^ |
| 3 | 141.7 | -2.0 | 141.8 | -0.8 | 141.6 | -1.4 |
| 4 | 139.9 | -1.7 | 141.0 | -0.7 | 140.4 | -1.1 |
| 5 | 138.4 | -1.3 | 140.4 | -0.5 | 139.4 | -0.9 |
| 6 | 137.2 | -1.0 | 140.0 | -0.4 | 138.6 | -0.7 |
| 7 | 136.4 | -0.7 | 139.7 | -0.2 | 138.0 | -0.4 |
| 8 | 135.9 | -0.4 | 139.5 | -0.1 | 137.7 | -0.2 |
| 9 | 135.6 | 0.0 | 139.5 | 0.0 | 137.6 | 0.0 |
| 10 | 135.8 | 0.3 | 139.6 | 0.2 | 137.7 | 0.2 |
| 11 | 136.2 | 0.6 | 139.9 | 0.3 | 138.1 | 0.5 |
| 12 | 137.0 | 0.9 | 140.3 | 0.5 | 138.7 | 0.7 |
| 13 | 138.0 | 1.2 | 140.8 | 0.6 | 139.5 | 0.9 |
| 14 | 139.4 | 1.6 | 141.5 | 0.7 | 140.6 | 1.2 |
| Abbreviations: POD, postoperative day | | | | | | |
| ^a^: Estimated sodium level is calculated from the linear mixed model as specified in supplementary methods. | | | | | | |
| ^b^: Estimated rate of change is derived from the corresponding slope of the linear mixed model throughout POD3 to POD14 | | | | | | |

**Table S3. Frequencies and Effect Sizes for Post-Surgical Serum Sodium Level and Other Conditions Following Per-Protocol (PP) Analysis.^a^**

|  | **Control** | **Strict Fluid Restriction** | | | | **Moderate Fluid Restriction** | | | | **Overall Fluid Restriction** | | | | **Overall** |
| --- | --- | --- | --- | --- | --- | --- | --- | --- | --- | --- | --- | --- | --- | --- |
|  | **(N=94)** | **(N=15)** | | | | **(N=14)** | | | | **(N=29)** | | | | **(N=123)** |
|  | *mean (SD)* | *mean (SD)* | *ED* | *95% CI* | *P value* | *mean (SD)* | *ED* | *95% CI* | *P value* | *mean (SD)* | *ED* | *95% CI* | *P value* | *mean (SD)* |
| **Serum sodium level (mEq/L)^b^** |  |  |  |  |  |  |  |  |  |  |  |  |  |  |
| Nadir^c^ | 135 (5.56) | 138 (2.67) | 3.50 | 1.68 — 5.31 | <0.001*** | 137 (4.42) | 1.60 | -1.14 — 4.34 | 0.24 | 137 (3.68) | 2.58 | 0.8 — 4.36 | 0.005** | 136 (5.28) |
| Daily average^d^ | 139 (3.09) | 140 (1.78) | 1.52 | 0.37 — 2.66 | 0.01* | 140 (2.09) | 1.11 | -0.22 — 2.45 | 0.10 | 140 (1.91) | 1.32 | 0.37 — 2.27 | 0.007** | 139 (2.90) |
|  | *no. of patients*  *(%)* | *no. of patients (%)* | *OR* | *95% CI* | *P value* | *no. of patients (%)* | *OR* | *95% CI* | *P value* | *no. of patients (%)* | *OR* | *95% CI* | *P value* | *no. of patients*  *(%)* |
| **Post-surgical conditions^e^** |  |  |  |  |  |  |  |  |  |  |  |  |  |  |
| Hyponatremia^f^ |  |  |  |  |  |  |  |  |  |  |  |  |  |  |
| Any severity | 30 (31.9%) | 1 (6.7%) | 0.15 | 0 — 1.11 | 0.06 | 2 (14.3%) | 0.36 | 0.04 — 1.77 | 0.22 | 3 (10.3%) | 0.25 | 0.04 — 0.91 | 0.03* | 33 (26.8%) |
| Mild hyponatremia | 18 (19.1%) | 1 (6.7%) | 0.30 | 0.01 — 2.26 | 0.46 | 1 (7.1%) | 0.33 | 0.01 — 2.46 | 0.46 | 2 (6.9%) | 0.32 | 0.03 — 1.46 | 0.15 | 20 (16.3%) |
| Moderate hyponatremia | 5 (5.3%) | 0 (0%) | 0.00 | 0 — 7.12 | >0.99 | 0 (0%) | 0.00 | 0 — 7.67 | >0.99 | 0 (0%) | 0.00 | 0.00 — 3.55 | 0.59 | 5 (4.1%) |
| Severe hyponatremia | 7 (7.4%) | 0 (0%) | 0.00 | 0 — 4.49 | 0.59 | 1 (7.1%) | 0.96 | 0.02 — 8.52 | >0.99 | 1 (3.4%) | 0.45 | 0.01 — 3.72 | 0.68 | 8 (6.5%) |
| Readmission |  | ` |  |  |  |  |  |  |  |  |  |  |  |  |
| All reasons | 14 (14.9%) | 0 (0%) | 0.00 | 0 — 1.79 | 0.21 | 3 (21.4%) | 1.51 | 0.24 — 6.81 | 0.69 | 3 (10.3%) | 0.65 | 0.11 — 2.58 | 0.76 | 17 (13.8%) |
| Due to Hyponatremia | 6 (6.4%) | 0 (0%) | 0.00 | — | >0.99 | 1 (7.1%) | 0.68 | 0.01 — 16.2 | >0.99 | 1 (3.4%) | 0.68 | 0.01 — 16.20 | >0.99 | 7 (5.7%) |
| *** *P*<.001, ***P*<.05, **P*<.1 | | | | | | | | | | | | | | |
| Abbreviations: ED, estimated difference; OR, odds ratio; 95% CI, 95% confidence interval | | | | | | | | | | | | | | |
| ^a^: Post-surgical sodium serum level and other conditions were reported for the 195 patients following pituitary surgery, stratified by randomization group. Analyses were conducted following PP approach, which only included only compliant patients in all three groups (All ad-lib patients are considered compliant). | | | | | | | | | | | | | | |
| groups with complete fluid intake data between POD3 and POD14. Compliance was defined as complying with their assigned protocol for ≥ 10 days between POD3 and POD14 | | | | | | | | | | | | | | |
| ^b^: Serum sodium level were reported by mean (SD). Estimated difference in serum sodium nadir and mean (along with associated 95% CI and P value) was calculated using unpaired T test for each fluid restriction group vs ad-lib group. | | | | | | | | | | | | | | |
| ^c^: Serum sodium nadir was defined as the lowest serum sodium level between POD3 and POD 14. | | | | | | | | | | | | | | |
| ^d^: Serum sodium daily average was calculated from taking the daily average of serum sodium level between POD3 to POD14. | | | | | | | | | | | | | | |
| ^e^: Post-surgical conditions were reported by no. of patients (%). Odds ratio of each condition (along with associated 96% CI and P value) was calculated using fisher's exact test for each fluid restriction group vs ad-lib group. | | | | | | | | | | | | | | |
| ^f^: Hyponatremia was defined as serum sodium concentration <135mEq/L. Mild hyponatremia: 130-135mEq/L; Moderate hyponatremia: 125-129mEq/L; Severe hyponatremia: <125mEq/L. | | | | | | | | | | | | | | |

**FIGURE LEGEND**

**Figure S1.** Flow chart of the Intention-to-treat analysis randomized population.

Comparison of longitudinal trend in fluid intake among patients in control, strict, and moderate FR groups from POD 3 to POD 14. To summarize the temporal trend graphically, a locally estimated scatterplot smoothing (LOESS) was applied. The shaded area represented the standard error of each curve.

**Figure S2.** Figure S2. Odds Ratio of Compliance to Fluid Restriction Protocol.

This forest plot shows the odds ratio of overall compliance, defined as complying for ≥ 10 days between the start of FR (POD 3) and the primary endpoint (POD 14). Odds ratios were calculated using logistic regression, with sex, mean thirst level, body mass index, AVP-D, and age as covariates. Thirst level was assessed on a scale from 1 to 5, with 5 as the most thirsty; mean thirst level was calculated from the mean thirst score from POD 3 to POD 14, with a mean score ≤ 3 defined as not thirsty in general, and a mean score > 3 as thirsty in general.

**Figure S1. Fluid Intake of Patients from POD 3 to POD 14**

**
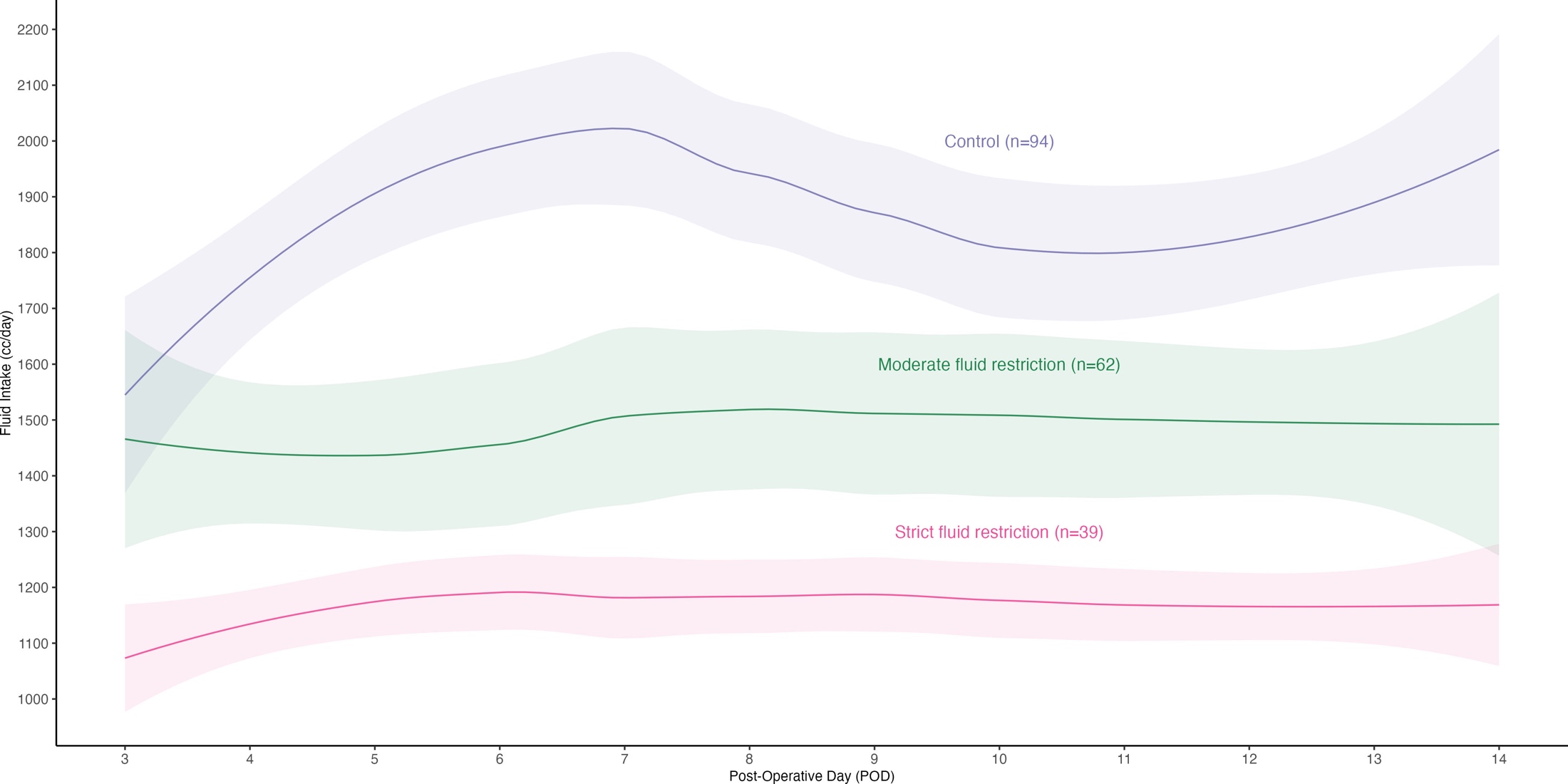
**

**Figure S2. Odds Ratio of Compliance to Fluid Restriction Protocol**
